# Excessive laxity of connective tissue in constipated children

**DOI:** 10.1038/s41598-022-05115-z

**Published:** 2022-01-19

**Authors:** Andrzej Załęski, Agnieszka Gawrońska, Piotr Albrecht, Marcin Banasiuk

**Affiliations:** 1 Department of Infectious Diseases, Tropical Diseases and Hepatology, Medical University of Warsaw, Warsaw, Poland; 2 Department of Pediatric Gastroenterology and Nutrition, Medical University of Warsaw, Warsaw, Poland; 3 Hospital for Infectious Diseases, Warsaw, Poland

**Keywords:** Gastrointestinal diseases, Constipation

## Abstract

Excessive laxity of the connective tissue refers to a group of inherited abnormalities manifested by disturbances in the functioning of internal organs, including the gastrointestinal tract. Increased susceptibility to stretching of the distal part of the large intestine and abnormal colonic motor function could explain the predisposition to the development of functional constipation in some children. Our aim was to determine whether patients with functional constipation are more likely to be characterized by congenital laxity of connective tissue compared to the population of healthy children. Children diagnosed with functional constipation according to the Rome III criteria were prospectively enrolled in the study (study group, S) and compared to otherwise healthy children (control group, C). Excessive laxity of the connective tissue was evaluated using the Beighton Score (BS) and expressed as median and interquartile range (IQR). The study included 411 patients (median age 7.8 years, min 3 years, max 18 years; 49% male), comprising 211 patients in the S group and 200 children in the C group. The median BS in the S group was significantly higher than in the C group (median: 5 points [IQR: 1–4.5] vs 2 points [IQR: 3–7], respectively; p = 0.000)*.* Furthermore, increased connective tissue laxity was observed more frequently in females (p < 0.05). Increased connective tissue laxity was more frequent in children with functional constipation, especially in girls. Excessive laxity of the connective tissue may be one of the etiological factors of functional constipation in children.

## Introduction

Excessive laxity of connective tissue is manifested by an increased range of motion in the joints above the accepted norms and disturbances in the functioning of organs composed of connective tissue^1,2^, including the gastrointestinal tract^1,3^. Its occurrence depends on many characteristics, such as gender, age, and race or ethnicity^3–5^. In Western countries, it affects approximately 10–35% of the population^1,4^, and is more prevalent in younger ages and in females^1,3,6–8^.

Excessive laxity of connective tissue often presents as generalized joint hypermobility (JH), and, in cases of the presence of musculoskeletal symptoms (mainly pain), joint hypermobility syndrome (JHS)^4,9^. Excessive laxity of connective tissue may occur in many genetic disorders, such as Down syndrome, *osteogenesis imperfecta*, and Marfan and Ehlers-Danlos syndromes^10,11^. Laxity of connective tissue should therefore be treated not as a separate disease syndrome, but as a pathological condition resulting from disturbances in the collagen structure due to assorted genetic backgrounds^10,12,13^. The Beighton Score (BS) is the basic tool for the assessment of JH^14^. This scale, especially when aided by a goniometer, is a reliable tool for the assessment of excessive laxity of connective tissue in adults and children^3,5^.

Functional disorders of the gastrointestinal tract are chronic, recurrent conditions that are not caused by biochemical or structural abnormalities. The most frequently observed disorders include regurgitation in infants (24.1%) and functional constipation in young children (18.5%) and young adults (14.1%)^15^. A possible relationship between gastrointestinal disorders (including functional constipation) and excessive laxity of connective tissue was recently advocated^1,3,4^. It is well recognized that motor activity of the gastrointestinal tract results from contraction and relaxation of the muscles of the longitudinal and transverse layers. These movements are supported by the tendon-like connective tissue net (TCTN) located between the muscle fibres. These components are also related to the connective-tissue plexus layer (CTPL)^16^. This mesh network plays an active role in coordinated contractile motor activity. Contraction of the circular muscle layer synchronously relaxes the longitudinal muscle, therefore disturbed connective tissue between both muscle layers may result in severe dysmotility presented as slow-transit constipation and dilation of the colon^16^.

Excessive laxity may result from disturbances in the proportions of type I collagen (thick collagen fibres) to type III (fine mesh fibres). In people with JH, the amount of type III collagen compared to type I collagen is higher than the norm^17^. Increased susceptibility to stretching of the wall of the distal colon and pelvic floor dysfunction may explain the predisposition to the development of functional constipation.

The aim of the present study was to determine whether patients with functional constipation were more likely to be characterized by laxity of connective tissue compared to the population of healthy children.

## Results

### Characteristics of population

We prospectively enrolled 411 children (median age 7.8 years, min 3 years, max 18 years; 49% male). The study group (S group) included 211 patients, while the control group (C group) included 200 children. The median BS for the whole sample was 4 (IQR: 2–6); 212 children (51.6%) presented BS ≥ 4 points, and 91 (22.1%) received BS scores ≥ 7 points. Clinical characteristics of participants are summarized in Table 1.

**Table 1:** Clinical characteristics of subjects.

Variable	S group (n = 211)	C group (n = 200)	p-value
Age (years), median (IQR)	7.7 (4.9–11)	7.8 (5.1–12)	0.486
Male gender, n (%)	104 (49.3)	98 (49)	0.953

*S* study group, *C* control group, *IQR* interquartile range.

### Evaluation of excessive laxity of connective tissue

**Figure 1:**
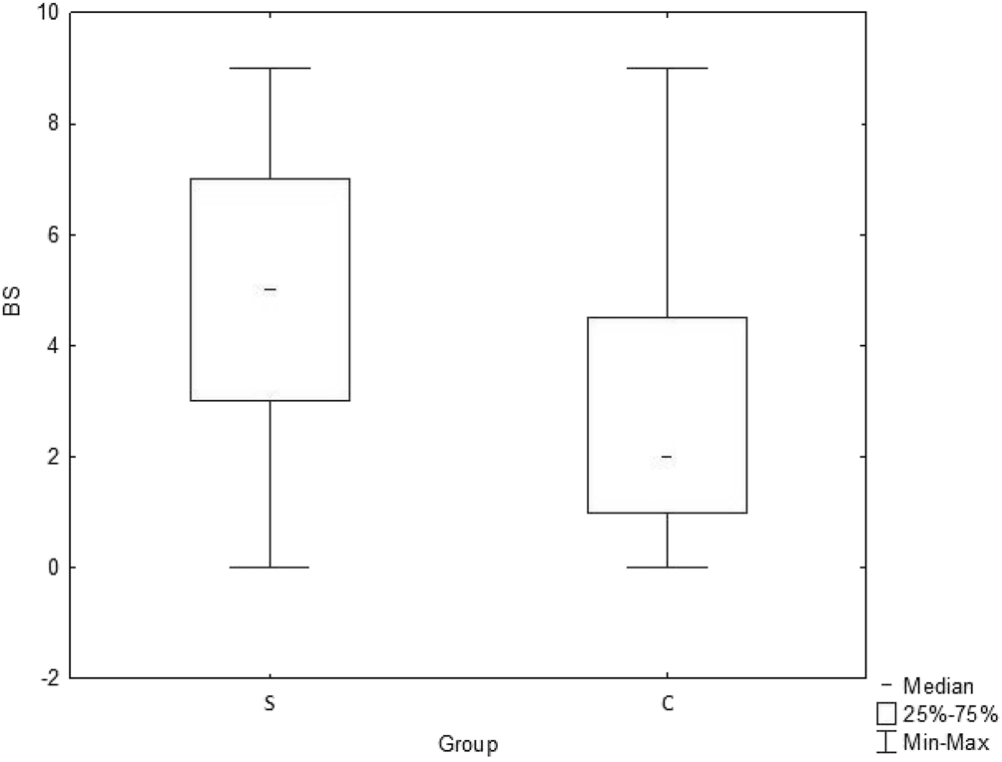
Median Beighton scores (BS) measured in both: study (S) and control (C) group.

The presence of connective tissue laxity as assessed by the BS was significantly greater in patients with functional constipation (S group) relative to participants without functional constipation (C group). The median BS in the S group was significantly higher than in the C group (median: 5 [IQR: 1–4.5] vs 2 [IQR: 3–7], respectively; p = 0.000). The ANOVA confirmed the results obtained in the Mann–Whitney U test (Fig. 1). Percentages of BS above the specific cut-off values in regard to the presence of constipation are summarized in Table 2.

**Table 2:** Percentages of Beighton score above the specific cut-off values in regard to the presence of constipation.

Cut-off value	S group	C group	p-value
BS ≥ 4 (n, %)	140/212 (66)	72/212 (34)	0.000
BS ≥ 7 (n, %)	66/91 (72.5.)	25/91 (27.5)	0.000

*S* study group, *C* control group, *BS* Beighton score.

### Excessive laxity of connective tissue in regard to gender

**Figure 2:**
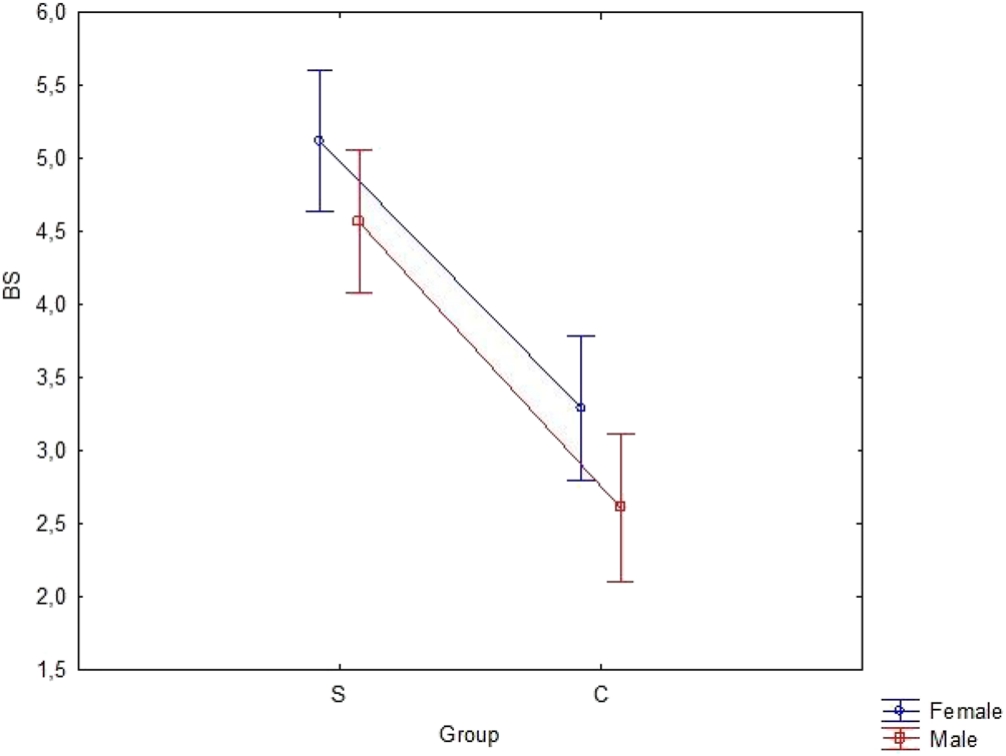
Lack of interaction between constipation and gender in regard to both: study (S) and ontrol (C) group.

The two-factor ANOVA tested three relationships concerning excessive laxity. The first test examined the specific effect of presence of constipation. The second test examined the specific effect of gender. The third test evaluated the interaction of constipation and gender. The results of the relationships between the factors and coexistence of functional constipation and excessive connective tissue laxity depending on gender are summarized in Table 3. The interaction between constipation and gender in regard to both groups is shown in Fig. 2. Gender was found to affect laxity as measured by BS. Table 4 summarizes the BS for each factor.

**Table 3:** The results of analysis of variance.

Effect	SS	*df*	MS	F	p-value
Constant	6241	1	6241	972.0	0.000
Group	367	1	367	57.2	0.000
Gender	39	1	39	6.1	0.014
Interaction	0	1	0	0.1	0.799
Error	2613	407	6		

*SS* sum-of squares, *df* degrees of freedom, *MS* mean squares.

**Table 4:** Beighton scores in regard to gender and the group (S—study group, C—control group).

Gender	Male			Female		
Group	S group	C group	p-value	S group	C group	p-value
BS median (IQR)	4 (2–7)	2 (1–4)	0.000	6 (3–7)	3 (1–5)	0.000

*S* study group, *C* control group, *BS* Beighton score, *IQR* interquartile range.

## Discussion

Our study found that children with functional constipation presented significantly more JH relative to otherwise healthy children. This finding suggests that excessive laxity of connective tissue, as measured by BS, may be one of the factors predisposing development of constipation in children.

The relationship between excessive laxity of connective tissue and disorders of the gastrointestinal tract—including inflammatory bowel diseases, coeliac disease, reflux, dyspepsia, postprandial fullness, complex pain related functional disorders, irritable bowel syndrome, and functional constipation (rectal evacuatory dysfunction)—has been reported in children and adults^1,3,18–23^. The BS was widely used to assess the laxity of connective tissue and proved to be a valuable tool^3,5^.

The results of our study indicate that, using a diagnostic criteria of BS ≥ 4, as many as 34% of Polish children without functional constipation may be diagnosed with excessive laxity of the connective tissue. Our results are in line with data published by Smits-Engelsmanet al., who reported 35% generalized hypermobility in a prospective survey of 551 children aged 6–12 years^5^. A slightly lower frequency (28.7%) was reported by Saps et al. in children aged 8–18 years, while Clinch et al. found a frequency of 19.2% in 14-year-old adolescents^24,25^. Van der Giessen et al. reported an incidence of up to 26.5% in children aged 4–9, but only 5% in children aged 10–12^26^. A substantially higher frequency (up to 55%) than in our study was reported by an Iranian study that evaluated children 3–10 years of age^27^. It must be noted that the original Beighton scale does not take into account differences between adults and children, gender, and inter-populational variability. Thus, discrepancies between studies might reflect not only different methodology (e.g., description of ranges of motion instead of goniometer measurement) but also potentially different characteristics of populations and samples. Moreover, the cut-off value for a positive test is widely discussed^1–3^. By changing the diagnostic criteria and increasing the cut-off point to 7 of 9 BS points in the Smits-Engelsman et al. study, JH was found in only 9% of patients^5^. Similarly, increasing the BS cut-off from 4 to 7 points in our study resulted in a diagnosis of JH in 27.5% of children. It is noteworthy that after increasing the cut-off score, the difference in frequency of JH between constipated and control groups was even more discernible (32% vs 45%, respectively). Therefore, in addition to identifying a significant relationship between laxity of connective tissue and functional constipation in children, this study contributes to the discussion regarding BS cut-off values. Further study is needed to identify different cut-off points in growing children relative to gender and age.

In our study, higher BS values were obtained in the female subgroup, supporting the findings of some studies^4,6^ but contrasting with others^5,7,26^. This discrepancy may reflect different age distributions between studies, as large cohort studies have suggested that younger age and gender correlated with higher incidence of hypermobility^7,8,14,28^. We also cannot rule out the influence of populational differences.

Several concepts have been proposed to explain the correlation between excessive laxity of connective tissue and constipation. Gastrointestinal motor activity is the result of contraction and relaxation of the muscles of the longitudinal and transverse layers. These movements are supported by the connective tissue network located between the muscle fibres. These components are also associated with the connective tissue plexus layer^16^. Meier-Ruge et al. revealed disturbances in the structure of connective tissue (lack of TCTN and/or CTPL) in children with constipation and normal innervation of the intestine^16,29^. These disturbances resulted in abnormal gut-muscle mechanics and slow-transit constipation with huge dilation of the colon^16,30^.

Disturbances in the proportions of type I collagen (thick collagen fibres) to type III (fine mesh fibres) in people with JH were also described^17^.A higher ratio of type III collagen to type I collagen might predispose to higher compliance of the large bowel, leading to ineffective motility and faecal impaction. Therefore, determining the spatial structure of connective tissue, as well as immunohistochemical proportions of collagen I, II and III, may serve as explanation of severe symptoms of constipation^1–4,17^. Genetic defects located on chromosomes 10, 11 and 16 were advocated as factors responsible for disturbances in collagen structure explaining familial occurrence of the disease described as desmosis coli^30–32^.

Apart from severe connective tissue abnormalities, other theories were proposed to describe the aetiology of symptoms. To avoid bothersome symptoms, children with urinary incontinence and JH may constantly use the pelvic floor muscles to compensate for the lack of supportive tissue, which may further lead to nonneurogenic bladder sphincter dysfunction, constipation, and faecal incontinence^33^. Petros and Swash proposed a theory focused on the musculo-elastic tissues within the pelvis and their role in anorectal dysfunction in adults^34^.The laxity of tissues may lead to the inability to generate appropriate intrarectal force during the bear-down manoeuvre. A greater prevalence of JH in adults with symptoms of rectal evacuatory dysfunction and significantly higher frequencies of morphological abnormalities in this group support this theory^23^.

Reilly et al. reported a higher frequency of generalized JH in a subgroup of boys with slow transit constipation, suggesting an influence of the disturbed connective tissue structure on the development of constipation^35^. In another small study on children with lower urinary tract symptoms, a higher proportion of boys with generalized JH had constipation when compared to control males, while no difference in frequency of constipation was found in females^33^. Similarly, in a large cohort study of children referred to a paediatric urology department due to voiding dysfunction, constipation was significantly more frequent in males with JH than in controls^36^. The results of these studies implicate a possible male gender predisposition; however, we could not provide support for this hypothesis in our cohort, as we did not find any correlation between gender and the presence of constipation in a large sample of children, regardless of urological problems. The inclusion criteria of the aforementioned studies differed from our study; thus, the smaller overall distribution of constipation as well as selection bias may explain a male gender predisposition.

In contrast to our results, other studies found no correlation between the presence of constipation and JH. Saps et al. reported that JH was equally prevalent in school children with and without functional gastrointestinal disorders, including functional constipation. Similar results were obtained by Khorasgani et al., who compared the frequency of JH in children with and without functional constipation^27^. In the latter study, JH was highly prevalent in both the study and control groups (58% vs 55% of children aged 3–10, respectively), which might explain the lack of correlation between hypermobility and constipation. The differences may reflect sample selection, namely the general population of school children in the first study or children referred to a paediatric clinic in second study. By contrast, our study used a tertiary centre cohort, which may be more likely to suffer from intractable constipation.

In sum, our study suggests that a thorough investigation should be performed in children with constipation referred to tertiary centres, as the condition may result from alterations in the biomechanics of connective tissue. We suggest that additional testing might be considered if JH is present in children with intractable constipation. However, our study has several limitations. First, controversies exist on both, Rome criteria and Beighton scale as being non-validated instruments. Despite the criticism, the latestguidelines of the European and North American Society for PediatricGastroenterology, Hepatology and Nutrition (ESPGHAN and NASPGHAN) claimsthat the Rome Criteriaserve as a reliabletool for making a diagnosis of functionaldisorders of the gastrointestinaltract in children. In 2016, afterapproval of the studyprotocol and whilepatientswereenrolled in the study, the ESPGHAN expertgrouppublishedan update of the Rome Criteria, namely version IV. Compared to the Rome III Criteria, the newiterationseparates the symptoms in childrenwhohave not learned to controltheirphysiologicalfunctions from thosewhohavealreadyacquiredthisskill. In the caseofolderchildren (i.e. ≥ 4 years of age), the onlychangewas the reduction of the duration of symptoms from 2 to 1 month (unification of the ESPGHAN and NASPGHAN guidelines). The changes in the Rome IV Criteria, in relation to the third edition, did not significantlychange the qualificationorcharacteristics of the patientsqualified for ourstudygroup. Lack of major changessuggest the reliability and supports the usage of the criteria in clinicaltrials. Similarily, Beightonscore, despitebeing a non-validatedtool, was widelyused in allrecentclinicaltrials, and the resultswerereportedin relation to controlresults. Therefore, we decided to usethisscale as moreinfomative and comparable with the results of others.

Second weakness is that, the stretching susceptibility of the digestive tract wall was not formally studied but inferred on the basis of the laxity of the connective tissue that builds the joints. In the literature, the assessment of joint laxity using the BS and a goniometer is considered a reliable method of assessing the general laxity of connective tissue^5^. Although the BS only assesses selected major joints, it has been shown that excessive laxity occurs simultaneously in other, unexplored joints^4,5^. Third, this study did not correlate the severity of constipation to the degree of laxity, which might further demonstrate the influence of laxity on the aetiology of constipation. Last, we did not perform additional tests, such as imaging of the rectum, to show greater distensibility or colonic transit time. In the future, the assessment of excessive laxity of connective tissue in patients with functional constipation based on manometric and histopathological examination of rectal specimens may be considered. However, these are invasive methods available in only a few specialized centres. Further, long-term observation of patients for the symptoms of constipation with the assessment of connective tissue laxity could confirm the relationship of these two diseases and justify further tests.

Connective tissue laxity in patients with functional constipation was found to be significantly different from patients without functional constipation. The study group with functional constipation exhibited significantly more frequent occurrence of connective tissue laxity than the control group. Disturbances in collagen structure may be one of the components contributing to the development of constipation in this group of patients. The study results indicate that gender should be considered as a factor influencing BS values. Future studies may find it worthwhile to determine the correlation of functional and structural tests of bowel function with excessive laxity of connective tissue in patients with functional constipation.

## Material and methods

### Participants

This was a prospective, cross-sectional, controlled study. Patients were recruited at the Department of Paediatric Gastroenterology and Nutrition at the Medical University of Warsaw, the Paediatric Gastroenterology Outpatient Clinic and the Department of Paediatrics, Clinical Decision Unit, Medical University of Warsaw.

The inclusion criteria for the study group (S) were as follows: (1) aged 3–18 years, (2) diagnosis of functional constipation according to the Rome III Criteria, (3) informed consent of parents or legal guardians for the child’s participation in the study. The exclusion criteria were as follows: (1) anatomical abnormalities: narrowing of the anus or rectum, as well as the condition after surgical correction of these abnormalities, (2) history of metabolic and gastrological disorders: hypothyroidism, hypercalcemia, hypokalaemia, cystic fibrosis, diabetes, celiac disease, (3) neuropathies: defects and injuries of the spinal cord, encephalopathies, (4) intestinal neuromuscular diseases: Hirschsprung’s disease, intestinal neural dysplasia, myopathies and visceral neuropathies, (5) abnormal abdominal muscles: Down syndrome, (6) drug use (opiates, phenobarbital, sucralfate, antidepressants, anticholinergics, sympathomimetic drugs, chemotherapy), and (7) in the care of a rheumatology or orthopaedic clinic.

The control group (C) consisted of children aged 3–18 years with no history of constipation or any chronic diseases whose legal guardians agreed to their participation in the study.

Before enrolling participants in the study, written informed consent from parents or legal guardians, as well as from participants who were over 16 years of age, was obtained.

The study was approved by the Biomedical Committee of the Medical University of Warsaw (KB/247/2014). The procedures used in this study adhere to the tenets of the Declaration of Helsinki.

Outcomemeasureswere as follows: (a) primaryoutcomemeasure: the comorbidity of joint hypermobility and functionalconstipation in children; (b) secondaryoutcomemeasure: the comorbidity of joint hypermobility and functionalconstipation, depending on gender.

### Procedure

Participants who fulfilled the inclusion criteria underwent physical examination by physicians trained in the assessment of connective tissue laxity. To prevent evaluation bias, the examiners did not know participants’ group assignment (study or control).

**Figure 3:**
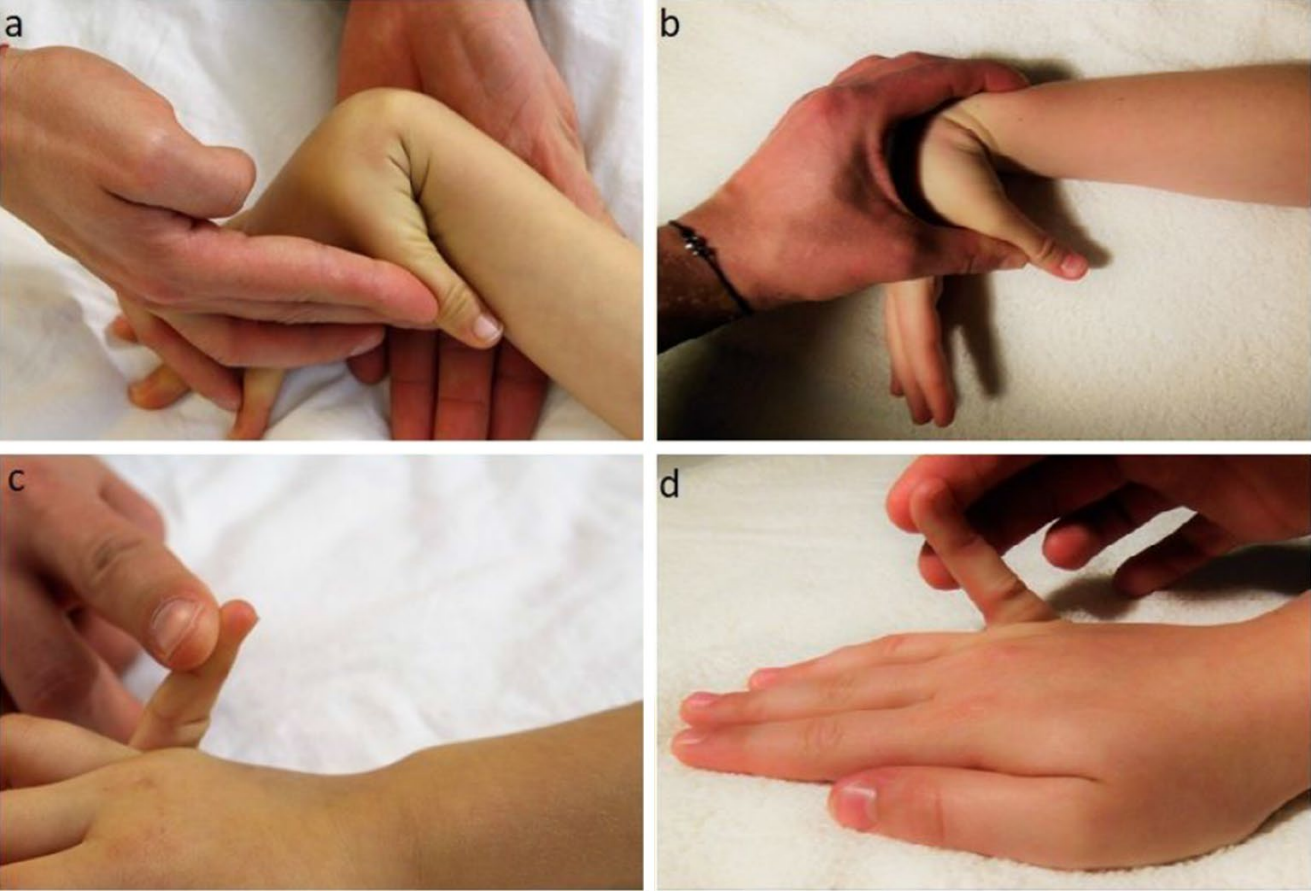
Examples of joint hypermobility: **(a,b)** excessive and normal passive apposition of thumb to forearm, **(c,d)** excessive and normal passive hyperextension of 5th metacarpophalangeal joint.

Children were assessed for the presence of connective tissue laxity on the basis of the BeightonScore (BS) (Table 5), using a goniometer and a standardized protocol. The protocol described the position of the patient’s body and the location of the goniometer, taking into account anatomical landmarks and the range of motion in the examined joint. Additionally, the protocol defined the scope of passive mobility in the examined joints in order to reduce error resulting from the patient’s belief about the possible range of motion. Children were tested in underwear (T-shirts, panties or shorts), without shoes. Before the assessment of laxity, the type and possible range of the tested movement was described verbally and demonstrated to the child by the examiner. Children were advised to relax their muscles as much as possible (as far as they could understand), and the movements were performed without causing pain. Nine measurements were performed for each participant using a 2-arm 360° goniometer (KaWeMedizintechnik, type V01, Horn Wellness Group, Germany). Participants’ ability to place a hand flat on the floor while bending forward with straight knees and to bend the thumb to the forearm were assessed without the use of measuring devices. Figure 3 shows several examples of joint hypermobility: (a–b) excessive and normal passive apposition of thumb to forearm, (c–d) excessive and normal passive hyperextension of 5th metacarpophalangeal joint.

**Table 5:** The 9-point Beighton score of hypermobility.

Specific joint laxity	Points	
	Right side	Left side
Passive apposition of thumb to forearm	1	1
Passive hyperextension of 5th MCP joint > 90°	1	1
Active hyperextension of elbow > 10°	1	1
Active hyperextension of knee > 10°	1	1
Ability to flex spine placing hands flat on the floor without bending knees	1	

*MCP* metacarpophalangealjoints.

### Sample size

Based on previous studies in children^35^, the sample size was calculated to detect an 18% difference in frequency of JH between groups. Assuming an *α* of 0.05, *β* of 0.05, drop-out rate of 20% and correction for continuity, the sample size was determined to be 418 children.

### Statistical methods

The distribution of variables was tested with Kolmogorov–Smirnov and Shapiro–Wilk tests. Chi-square tests were used to evaluate differences in frequencies. Since the Beighton scale used to measure laxity is an ordinal scale, non-parametric tests were used in the analysis. The Mann–Whitney U test was used to evaluate significant differences between groups in terms of laxity, without gender division, as well as separately for boys and girls. To reduce the possibility of making interpretation errors, a one- and a two-way ANOVA were performed to examine laxity (as measured by BS) relative to presence of constipation and/or sex differences. The null hypothesis was verified on the basis of the Fisher-Snedecor statistics. The homogeneity of variance was verified using the Levene and Bartlett tests based on the F statistic (in the case of the first two tests). ANOVA is relatively insensitive to the violation of the normality of the random component distribution, and the obtained statistics have an asymptotic normal distribution by virtue of the central limit theorem. Thus, the use of one-way ANOVA was justified. Statistical significance was defined as a p < 0.05. Statistica 13 (Statsoft, Oklahoma, USA) was used for all analyses.

## Data Availability

All data are available upon request from corresponding author.
